# Significance of *Aurora B *overexpression in hepatocellular carcinoma. *Aurora B *Overexpression in HCC

**DOI:** 10.1186/1471-2407-10-461

**Published:** 2010-08-28

**Authors:** Zhong-Zhe Lin, Yung-Ming Jeng, Fu-Chang Hu, Hung-Wei Pan, Hsin-Wei Tsao, Po-Lin Lai, Po-Huang Lee, Ann-Lii Cheng, Hey-Chi Hsu

**Affiliations:** 1 Departments of Oncology, National Taiwan University Hospital Yun-Lin Branch, No. 360, Singjhong Village, Huwei Township, Yun-Lin County, 63252, Taiwan; 2 National Center of Excellence for Clinical Trial and Research, National Taiwan University Hospital, No. 7, Chung-Shan S. Rd., Taipei, 10016, Taiwan; 3 Department of Pathology, National Taiwan University Hospital, No. 7, Chung-Shan S. Rd., Taipei, 10016, Taiwan; 4 Department of Medical Research, National Taiwan University Hospital, No. 7, Chung-Shan S. Rd., Taipei, 10016, Taiwan; 5 Department of Surgery, National Taiwan University Hospital, No. 7, Chung-Shan S. Rd., Taipei, 10016, Taiwan; 6 Department of Oncology, National Taiwan University Hospital, No. 7, Chung-Shan S. Rd., Taipei, 10016, Taiwan; 7 Department of Internal Medicine, National Taiwan University Hospital, No. 7, Chung-Shan S. Rd., Taipei, 10016, Taiwan; 8 Graduate Institute of Oncology, National Taiwan University College of Medicine, No.1, Jen-Ai Rd. Section 1, Taipei, 10051, Taiwan

## Abstract

**Background:**

To investigate the significance of Aurora B expression in hepatocellular carcinoma (HCC).

**Methods:**

The *Aurora B *and *Aurora A *mRNA level was measured in 160 HCCs and the paired nontumorous liver tissues by reverse transcription-polymerase chain reaction. Mutations of the *p53 *and *β-catenin *genes were analyzed in 134 and 150 tumors, respectively, by direct sequencing of exon 2 to exon 11 of *p53 *and exon 3 of *β-catenin*. Anticancer effects of AZD1152-HQPA, an Aurora B kinase selective inhibitor, were examined in Huh-7 and Hep3B cell lines.

**Results:**

*Aurora B *was overexpressed in 98 (61%) of 160 HCCs and in all 7 HCC cell lines examined. The overexpression of *Aurora B *was associated with *Aurora A *overexpression (*P *= 0.0003) and *p53 *mutation (*P *= 0.002) and was inversely associated with *β*-*catenin *mutation (*P *= 0.002). *Aurora B *overexpression correlated with worse clinicopathologic characteristics. Multivariate analysis confirmed that *Aurora B *overexpression was an independent poor prognostic factor, despite its interaction with Aurora A overexpression and mutations of *p53 *and *β*-*catenin*. In Huh-7 and Hep3B cells, AZD1152-HQPA induced proliferation blockade, histone H3 (Ser10) dephosphorylation, cell cycle disturbance, and apoptosis.

**Conclusion:**

*Aurora B *overexpression is an independent molecular marker predicting tumor invasiveness and poor prognosis of HCC. Aurora B kinase selective inhibitors are potential therapeutic agents for HCC treatment.

## Backgroud

Hepatocellular carcinoma (HCC) is the leading cause of cancer mortality in Taiwan [1] and many other countries in Asia and Africa [2]. The incidence of HCC is increasing in Europe and the United States [3]. In 2002, HCC became the sixth most common cancer worldwide with 626,000 annual new cases [4]. Despite surgical resection, which provides an opportunity for cure, the majority of patients with HCC have a dismal prognosis [5] because tumor recurrence frequently develops and usually leads to patient's mortality [6]. The development of HCC is closely related to chronic hepatitis B or C, cirrhosis of any etiology, and aflatoxin B1 exposure [2]. However, the detailed molecular mechanisms of hepatocarcinogenesis are still not fully understood [7]; molecular factors capable of predicting clinical outcome of HCC and acting as potential therapeutic targets remain limited. The identification of molecular markers related to hepatocarcinogenesis, tumor progression, and poor clinical outcome would benefit patients, providing for better management planning and serving as potential therapeutic targets for novel HCC drug treatments.

Genomic instability has been correlated with hepatocarcinogenesis [8], and increased chromosomal instability has been associated with differentiation status of human HCC [9]. Aurora kinases, a subfamily of serine/threonine mitotic kinases, are thought to be key molecules required for maintaining accurate cell cycling and genomic stability [10]. We previously showed that *Aurora A *was overexpressed in 137 (61%) of 224 human HCCs and that the overexpression of *Aurora A *was associated with aggressive tumor characteristics and poor prognosis of patients [11]. Furthermore, we demonstrated that VE-465, a novel pan-Aurora kinase inhibitor, had anticancer effects in preclinical models of human HCC [12]. These findings indicated that Aurora kinases may be important biomarkers and potential therapeutic targets in HCC.

There are three highly related Aurora kinases in mammals, Aurora A, B, and C. Aurora A and Aurora B share a high degree of sequence homology in their catalytic domains, and overexpression of each has been identified in many human cancers [13].

Despite their sequence similarity, Aurora A and Aurora B differ in chromosomal gene loci, subcellular localization, cellular functions, and signaling substrates [13]. The Aurora A kinase gene is localized to chromosome 20q13.2, and that for Aurora B kinase is localized to chromosome 17p13.1. Aurora A kinase protein is localized in the centrosome and spindle poles and plays important roles in centrosome maturation and spindle assembly [14]. Aurora B kinase, which is a chromosome passenger protein localized in the centromeres during early mitosis and then at the spindle midzone after anaphase, is essential for chromosome biorientation, function of the spindle assembly checkpoint, and cytokinesis [15]. The enthusiasm of exploring Aurora kinases as anticancer therapeutic targets initially centered on Aurora A, but recent studies have demonstrated that several Aurora kinase inhibitors exhibit anticancer activity resembling that of Aurora B disruption induced by genetic methods [16]. Therefore, determination of the distinctive roles in carcinogenesis and individual clinical significance of Aurora A and Aurora B is mandatory. The aims of this study were to elucidate the clinicopathologic significance of *Aurora B *expression and *Aurora A *expression in HCC and to correlate their expression with *p53 *and *β-catenin *mutations, the two most frequently mutated genes in HCC [7,11].

## Methods

### Tissue samples

During the period January 1987 through December 1997, 160 surgically resected, primary unifocal HCCs were selected for this study. After resection, tumor tissues were immediately cut into small pieces, snap frozen in liquid nitrogen, and stored in deep freezer. Patients had received comprehensive pathologic assessment and regular follow-up at National Taiwan University Hospital, as described previously [17,18]. This study was compliant with the regulations of the Ethics Committee of the host institution. The 160 patients included 122 men and 38 women with a mean age of 57 years (range, 14-88 years). Serum hepatitis B surface antigen (HBsAg) was detected in 107 cases (67%) and antihepatitis C virus antibody in 53 (35%), including 13 positive for both. Elevated α-fetoprotein (AFP; ≥200 ng/mL) was detected in 80 cases (50%). Liver cirrhosis was found in 61 patients (38%). All patients had adequate liver function reserve at the time of surgery. None of the patients had received local or systemic therapy before surgery.

### Histological study and tumor staging

Tumor grade was divided into 3 groups: well-differentiated (grade I, 31 cases), moderately differentiated (grade II, 74 cases), and poorly differentiated (grade III-IV, 55 cases). The unifocal HCC was staged as stages I, II, IIIA, IIIB, and IV, as described previously [11,19,20]. Staging was based on the International Union Against Cancer criteria, with slight modification because HCC tends to spread in the liver via vascular invasion, which is an important unfavorable prognostic factor for this disease [21]. Stage I HCC included tumors that were ≤ 2 cm and showed no evidence of liver and vascular invasion (4 cases). Stage II HCCs included tumors that were ≤ 2 cm for which vascular invasion was limited to small vessels in the tumor capsule, as well as encapsulated tumors > 2 cm with no evidence of liver or vascular invasion (62 cases). Stage IIIA HCCs included invasive tumors > 2 cm with invasion of small vessels in the tumor capsule and/or satellites near the tumor, but no portal vein invasion (25 cases). Stage IIIB HCCs included tumors with invasion of the portal vein branch near the tumor, but not of the distant portal vein in the liver parenchyma (23 cases). Stage IV included tumors with involvement of major portal vein branches, satellites extending deeply into the surrounding liver, tumor rupture, or invasion of the surrounding organs (46 cases). No evidence of regional lymph node or distant metastasis was noted at the time of surgery in any of the cases. Among the 160 patients studied, 149 were eligible for the evaluation of early tumor recurrence (ETR; ≤12 months). Eleven patients who died within 1 year after surgery without objective evidence of tumor recurrence were excluded from the evaluation of ETR.

### Reverse transcription-polymerase chain reaction

Reverse transcription-polymerase chain reaction (RT-PCR) was used to determine the mRNA levels of *Aurora A *and *Aurora B *in paired HCCs and nontumorous liver samples, as described elsewhere [11,22]. The ribosomal protein *S26 *mRNA, a housekeeping gene, was used as an internal control [23]. Briefly, total RNA was isolated from the frozen tissues using a guanidium isothiocyanate/CsCl method. RNA was quantified by spectrophotometry at 260 nm. Stock RNA samples were kept in alcohol in deep freezer until used. Complementary DNA (cDNA) was prepared from the total RNA of paired HCCs and nontumorous liver samples. Two microliter reverse transcription product, 1.25 units Pro Taq polymerase (Protech Technology Enterprise, Taipei, Taiwan), Pro Taq buffer, and 200 μM dATP, dCTP, dGTP, and dTTP (each) were mixed with primer pairs for *Aurora A, Aurora B*, and *S26 *in a total volume of 30 μl. One-tube PCR reaction was stopped at the exponential phase of gene amplification: 29 cycles for *Aurora A*, 32 for *Aurora B*, and 23 for *S26*. The reaction was performed in an automatic DNA thermal cycler (model 480; Perkin-Elmer Co., Wellesley, MA), with limited reaction reagents (Tag enzyme and dNTPs), and processed with initial heating at 94°C for 2 minutes, followed by 29 (*Aurora A*) or 32 (*Aurora B*) PCR reaction cycles of 94°C for 30 seconds, annealing at 60°C for 1 minute, extension at 72°C for 1 minute, and a final 72°C extension for 10 minutes. The PCR reaction was stopped at cycle 7 (*Aurora A*) or 10 (*Aurora B*), and the reaction tubes were quenched on ice to allow adding *S26 *primers, then complete the final 23 PCR reaction cycles. Primers for amplified genes were as follows: *Aurora A*-F (AATTGCAGATTTTGGGTGGT), *Aurora A*-R (AAACTTCAGTAGCATGTTCCTGTC), *Aurora B*-F (ATCGTGGCGCTCAAGGTCCT), *Aurora B*-R (GATGCACTCTCAAAGGGTGGG), *S26*-F (CCGTGCCTCCAAGATGACAAAG), *S26*-R (GTTCGGTCCTTGCGGGCTTCAC). The PCR products were electrophoresed on a 2% agarose gel. Concentrations of the PCR fragments were determined with the IS-1000 digital imaging system (Alpha Innotech, San Leandro, CA). The *Aurora A *and *Aurora B *mRNA levels were determined according to the ratio of signal intensity for *Aurora A *or *B *to that of *S26 *as measured by 1 D Image Analysis software (Kodak Digital Science, Rochester, NY) and scored as high (ratio >1.0), moderate (ratio > 0.5 and ≤1.0), or low (ratio ≤0.5). The *Aurora A *and *Aurora B *mRNA levels of nontumorous liver rarely exceeded a ratio of 0.5, and a ratio > 0.5 was regarded as overexpression.

### Analysis of p53 and β-catenin mutations

Mutations of the *p53 *and *β-catenin *genes were analyzed in 134 and 150 tumors, respectively, by direct sequencing of exon 2 to exon 11 of *p53 *and exon 3 of *β-catenin*, as described previously [17,24,25]. Samples with incomplete study results were excluded from statistical analysis.

### Follow-up observation and assessment of early tumor recurrence

Early tumor recurrence (ETR) was designated as intrahepatic tumor recurrence or distant metastasis detected by imaging tools, pathology and/or high AFP levels within 12 months. All 160 patients had been followed for more than 5 years or until death. At the end of the follow-up in November 2008, 37 patients remained alive. One hundred forty-nine cases (93%) were eligible for evaluation of ETR.

Seventy-three (46%) cases had ETR. Among the 73 patients, 11 (15%), 26 (36%), 7 (10%), 1 (1%) received tumor resection, transhepatic arterial embolization, chemotherapy, or radiotherapy, respectively. Fifty-one (32%) of the 160 cases had late tumor recurrence more than 12 months after the initial hepatectomy. Among the 51 patients, 18 (35%), 24 (47%), 4 (8%), 2 (4%) received tumor resection, transhepatic arterial embolization, chemotherapy, or radiotherapy, respectively.

### Cell culture and reagents

The liver cancer cell lines Huh-7, HepG2, Hep3B, PLC5, HCC36, HA59T, SK-hep-1, and Tong were cultured in Dulbecco's modified Eagle's medium (DMEM) plus 10% fetal bovine serum (FBS), supplemented with penicillin and streptomycin. Cells were maintained in a humidified incubator with 5% CO_2 _in air at 37°C.

AZD1152-HQPA is a selective inhibitor of Aurora B (inhibition constant Ki, 0.36 nM) compared with Aurora A (inhibition constant Ki, 1369 nM) [26]. AZD1152-HQPA, provided by AstraZeneca Pharmaceuticals (Macclesfield, UK), was used for *in vitro *cell line studies.

### Western blot analysis

Western blotting was performed as described previously [11]. The following primary antibodies were used: anti-Aurora B (Novus Biologicals, Littleton, CO, USA), anti-Aurora A (Novus Biologicals), anti-α-tubulin antibody (Sigma-Aldrich, St. Louis, MO, USA), anti-phosphorylated histone H3 (Ser10) (Santa Cruz Biotechnology, Inc., Santa Cruz, CA, USA), and anti-phosphorylated Aurora A (T288) (Cell Signaling Technology, Inc., Danvers, MA, USA). The final images were developed with a chemiluminescence reagent.

### Cell viability and flow cytometry

A total of 5 × 10^4 ^Huh-7 or Hep3B cells were plated in six-well plates. After overnight culture, cells were treated with DMSO or 1, 5, 25, and 125 nM of AZD1152-HQPA. At 72 hours of drug treatment, cells were trypsinized and the total number of cells were counted using hemocytometer. Trypan blue dye exclusion assay was used to determine the number of viable cells. The experiments were carried out in 3-4 replicates and repeated trice.

Cells in logarithmical growth were incubated with either AZD1152-HQPA or DMSO for 24 to 48 hours. Cells were labeled with 0.5~1 mL propidium iodide (50 μg/mL) after being trypsinized and fixed in 70% methanol overnight. Cell cycle profiles and sub-G1 fractions were determined using a FACS caliber (Becton Dickinson, San Jose, CA, USA).

### Statistical analysis

Data analyses were carried out with Statistical Analysis System software (version 9.1; SAS Institute, Inc., Cary, NC). Two-tailed *P *< 0.05 was considered statistically significant. The χ^2^, Fisher's exact test, and log-rank test were used for univariate analyses. Multivariate analyses were conducted for ETR, tumor size, stage, and grade by fitting multiple logistic regression models [27]. Time to death was analyzed by fitting multiple Cox's proportional hazards models [28]. In our regression analyses, basic model-fitting techniques for (*a*) variable selection, (*b*) goodness-of-fit assessment, and (*c*) regression diagnostics (including residual analysis, influence analysis, and check of multicollinearity) were used to ensure the quality of the analysis results [27,28].

For the *in vitro *studies, the mean differences among groups were tested by one-way analysis of variance (ANOVA) followed by multiple comparisons using the Dunnett's post hoc test or the Bonferroni's correction of alpha level.

## Results

### Expression of *Aurora B *mRNA and protein in liver and hepatocellular carcinoma

**Figure 1:**
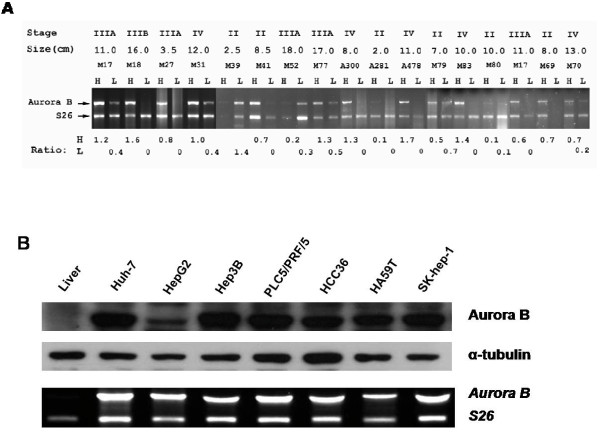
**Overexpression of Aurora B in hepatocellular carcinoma**. (A) *Aurora B *mRNA expression in paired hepatocellular carcinoma (H) and nontumorous liver parenchyma (L) was measured by quantitative RT-PCR in the exponential phase of amplification. Overexpression of *Aurora B *(*Aurora B*/*S26 *ratio > 0.5) was detected in 9 of 13 representative tumor samples and in 2 nontumorous liver tissues. Tumor stage and tumor size are depicted above. (B) Protein lysates and mRNA from nontumorous liver tissues and 7 liver cancer cell lines were analyzed by immunoblotting and quantitative RT-PCR. All 7 liver cancer cell lines show overexpression of Aurora B at protein level (*top panel*). The mRNA expressions were correlated with protein levels (*bottom panel*).

Using RT-PCR in the linear range, *Aurora B *mRNA overexpression was detected in 98 (61%) of 160 surgically resceted, primary unifocal HCC specimens (Fig. 1A). Of these 160 HCCs, RNA samples of nontumorous liver were examined in 153 cases. In nontumorous liver, overexpression of *Aurora B *mRNA at a moderate level was detected in 2 cases (1.3%). We then examined *Aurora B *gene expression in cell lines, and all 7 liver cancer cell lines showed high expression levels of *Aurora B *mRNA, which correlated with protein levels (Fig. 1B).

### Clinicopathologic significance of *Aurora B *mRNA overexpression in hepatocellular carcinoma

To elucidate the biologic significance of Aurora B in HCC, we correlated *Aurora B *expression with major clinicopathologic features of HCC. As shown in Table 1, *Aurora B *overexpression was associated with high serum AFP level (≥200 ng/mL; *P *< 0.0001), but not with age, gender, chronic hepatitis B/C virus infection, or liver functional reserve (Child-Pugh class).

**Table 1:** Correlation of *Aurora B *mRNA expression with clinicopathologic and molecular features in 160 patients with primary unifocal HCC by univariate logistic regression analyses

Feature	*Aurora B *overexpression			
	
	Total	*n *(%)	OR (95% CI)*	*P *value
Age				
> 55	88	49 (56)	1.0	
≤55	72	49 (68)	1.7 (0.89-3.25)	0.11
Gender				
Female	38	23 (61)	1.0	
Male	122	75 (61)	1.04 (0.49-2.19)	0.916
HBsAg				
Negative	53	27 (51)	1.0	
Positive	107	71 (66)	1.9 (0.97-3.72)	0.06
Anti-HCV				
Negative	100	68 (68)	1.0	
Positive	53	30 (57)	0.8 (0.41-1.57)	0.517
AFP				
< 200	80	33 (41)	1.0	
≥200	80	65 (81)	6.17 (3.01-12.63)	< 0.0001
Child-Pugh				
A	147	93 (63)	1.0	
B	11	5 (45)	0.48 (0.14-1.66)	0.24
Cirrhosis				
No	99	66 (67)	1.0	
Yes	61	32 (52)	0.55 (0.29-1.06)	0.073
Size (cm)				
≤5	72	37 (51)	1.0	
> 5	88	61 (69)	2.14 (1.12-4.08)	0.021
Grade				
I	31	10 (32)	1.0	
II	74	48 (65)	3.88 (1.59-9.46)	
III-IV	55	40 (73)	5.6 (2.15-14.61)	0.0007
Stage				
I-II	66	21 (32)	1.0	
IIIA-IV	94	77 (82)	9.71 (4.64-20.3)	< 0.0001
ETR†				
No	76	28 (37)	1.0	
Yes	73	61 (84)	8.71 (4.02-18.91)	< 0.0001
5-year survival				
Yes	56	22 (39)	1.0	
No	104	76 (73)	4.19 (2.11-8.36)	< 0.0001
*Aurora A *overexpression				
No	60	26 (43)	1.0	
Yes	100	72 (72)	3.36 (1.72-6.58)	0.0003
*p53 *mutation				
No	68	34 (50)	1.0	
Yes	66	50 (76)	3.13 (1.5-6.53)	0.002
*β-catenin *mutation				
No	128	85 (66)	1.0	
Yes	22	7 (32)	0.24 (0.09-0.62)	0.002

*OR: odds ratio; CI: confidence interval

†ETR: early tumor recurrence within 12 months after surgery

Histologically, *Aurora B *overexpression did not correlate with the presence of liver cirrhosis. Nevertheless, HCCs with *Aurora B *overexpression were associated with large tumor size (> 5 cm; *P *= 0.021), high-grade histology (*P *= 0.0007), and advanced tumor stage (*P *< 0.0001).

Genes for *p53*, *β-catenin*, and *Aurora A *are most frequently deregulated in HCC and are closely associated with HCC progression [7,11]. Therefore, relations between *Aurora B *overexpression with mutations of *p53 *and *β-catenin *and with *Aurora A *overexpression were analyzed. Table 1 shows that *Aurora B *overexpression was correlated with *Aurora A *overexpression (*P *= 0.0003) and *p53 *mutation (*P *= 0.002). In contrast, *Aurora B *was more frequently overexpressed in HCCs without *β-catenin *mutation (*P *= 0.002).

### *Aurora B *overexpression predicts early tumor recurrence and poor prognosis

**Figure 2:**
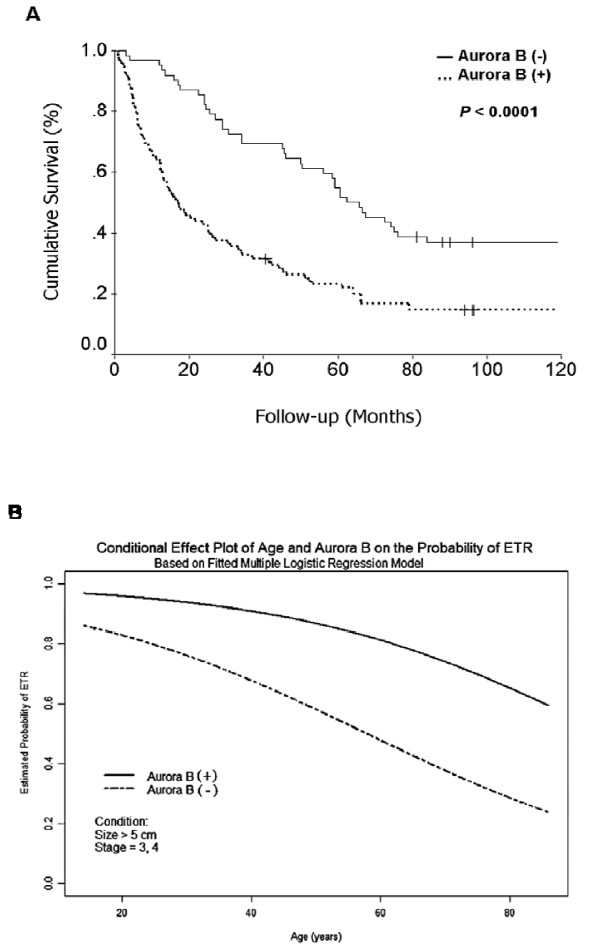
***Aurora B *overexpression predicts early tumor recurrence and poor prognosis**. (A) Cumulative survival curve for 160 patients with primary unifocal hepatocellular carcinoma (HCC). HCC with *Aurora B *mRNA overexpression, designated Aurora B (+), had significantly worse 5-year survival than HCC without *Aurora B *mRNA overexpression, designated Aurora B (-). (B) Conditional effect plot of age on ETR with or without overexpression of *Aurora B *mRNA. Based on multiple logistic regression model (n = 149). The other independent variables were fixed as size > 5 cm and stage III-IV.

HCC with *Aurora B *overexpression were associated with worse 5-year survival than HCC without *Aurora B *overexpression (*P *< 0.0001; Table 1 and Fig. 2A). Moreover, HCC with *Aurora B *overexpression showed more frequent ETR (*P *< 0.0001; Table 1), the most crucial clinical event associated with poor prognosis of HCC after hepatectomy [6,19]. As listed in Table 2, multivariate analysis showed that *Aurora B *overexpression (odds ratio [OR], 4.679; *P *= 0.0011], tumor size (OR, 3.735; *P *= 0.0031), tumor stage (OR, 3.611; *P *= 0.0073), and age ≤55 years (OR, 1.043; *P *= 0.0245) were significant independent risk factors by Cox's proportional hazards model for the occurrence of ETR. A conditional effect plot of age and *Aurora B *overexpression on ETR was drawn based on the multiple logistic regression model with fixed tumor size and stage (Fig. 2B). The probability of ETR was significantly higher in patients with HCC showing *Aurora B *overexpression. Furthermore, ETR (OR, 29.181; *P *< 0.0001), tumor grade (OR, 1.516; *P *= 0.0041), and tumor size (OR, 1.072; *P *= 0.0048) were significant independent risk factors associated with poor patient survival (Table 2). Principally, we found that *Aurora B *overexpression was an independent risk factor associated with high-stage tumor (OR, 7.439; *P *= 0.0003; Table 2) and ETR (OR, 4.679; *P *= 0.0011), hence contributing to poor patient survival. Nevertheless, *Aurora B *overexpression did not exert prognostic effects for tumor size or tumor grade (Table 2).

**Table 2:** Multivariate analyses of the risk factors associated with ETR, tumor size, tumor stage, tumor grade, and survival of the patients with primary unifocal HCC

Covariate	Variable Estimate	SE	**Wald χ**^**2**^	*P*	OR
ETR (yes) **^a^**					
Intercept	-0.2071	1.1146	0.0345	0.8526	--
*Aurora B *↑*	1.543	0.4721	10.6826	0.0011	4.679
Size	1.3177	0.4451	8.7658	0.0031	3.735
Stage	1.2839	0.4789	7.1876	0.0073	3.611
Age ≤55	0.0414	0.0184	5.0581	0.0245	1.043
Size ^b^					
Intercept	-0.7535	0.4762	2.5037	0.1136	--
*p53 *mutation	1.5197	0.4828	9.9073	0.0016	4.571
*Aurora A *↑	1.1577	0.4937	5.48	0.0192	3.176
HCV	-1.0277	0.4958	4.2961	0.0382	0.358
Cirrhosis	-1.1187	0.4989	5.0291	0.0249	0.327
Stage III-IV ^c^					
Intercept	-4.3949	1.1039	15.8508	< 0.0001	--
*Aurora B *↑	2.0067	0.5516	13.2351	0.0003	7.439
*p53 *mutation	1.0769	0.5399	3.9782	0.0461	2.936
Grade	1.0442	0.3965	6.9351	0.0085	2.841
Size	0.2495	0.0755	10.9173	0.001	1.283
Grade ^d^					
Intercept	0.4449	0.4037	1.2146	0.2704	--
HBV	0.8776	0.3814	5.2947	0.0214	2.405
*p53 *mutation	0.8058	0.3677	4.8033	0.0284	2.239
AFP	0.8056	0.3808	4.4754	0.0344	2.238
*β-catenin *mutation	-1.4923	0.5578	7.1569	0.0075	0.225
Survival time					
ETR	3.3735	0.5685	35.2186	< 0.0001	29.181
Grade	0.4158	0.1448	8.2453	0.0041	1.516
Size	0.0698	0.0247	7.9621	0.0048	1.072

^a^Logistic regression model: n = 149, percentage of concordant pairs = 86.5%, percentage of discordant pairs = 13.3%, adjusted generalized R^2 ^= 0.3755, Hosmer and Lemeshow goodness-of-fit test *P *= 0.4583 (degrees of freedom = 8).

^b^Logistic regression model: n = 120, percentage of concordant pairs = 84.3%, percentage of discordant pairs = 14.4%, adjusted generalized R^2 ^= 0.3507, Hosmer and Lemeshow goodness-of-fit test *P *= 0.3603 (degrees of freedom = 8).

^c^Logistic regression model: n = 125, percentage of concordant pairs = 90.9%, percentage of discordant pairs = 8.9%, adjusted generalized R^2 ^= 0.4647, Hosmer and Lemeshow goodness-of-fit test *P *= 0.7933 (degrees of freedom = 8).

^d^Logistic regression model: n = 123, percentage of concordant pairs = 65.8%, percentage of discordant pairs = 21.6%, adjusted generalized R^2 ^= 0.2291, Hosmer and Lemeshow goodness-of-fit test *P *= not applicable.

*↑, mRNA overexpression

### Interaction of *Aurora B *overexpression with *Aurora A *overexpression and mutations of p53 and β-catenin in hepatocellular carcinoma

Because both Aurora A and Aurora B correlate closely with unfavorable prognosis of HCC and may be potential therapeutic targets [11,12], we analyzed the possible interplay between these two important biomarkers. In this study, *Aurora A *overexpression, which was found in 100 (63%) of 160 HCCs examined, significantly correlated with *Aurora B *overexpression (*P *= 0.0003; Table 1). Moreover, as shown in Table 3, HCC with overexpression of both *Aurora A *and *Aurora B *showed the highest occurrence of high serum AFP level (≥200 ng/mL; 71%), large tumor size (> 5 cm; 72%), grade II to IV tumor (94%), stage IIIA to IV tumor (82%), *p53 *mutation (64%), wild-type *β-catenin *(92%), and the worst 5-year survival rate (19%) than the other groups.

**Table 3:** Cooperations of *Aurora B *and *Aurora A *mRNA expressions in relation to clinicopathologic and molecular features in 160 patients with primary unifocal HCC

Feature	*Aurora B */ *Aurora A *expression number (%)				*P *value
	+/+ (*n *= 72)	+/- (*n *= 26)	-/+ (*n *= 28)	-/- (*n *= 34)	
AFP					< 0.0001
< 200	21 (29)	12 (46)	18 (64)	29 (85)	
≥200	51 (71)	14 (54)	10 (36)	5 (15)	
Size (cm)					< 0.0001
≤5	20 (28)	17 (65)	11 (39)	24 (71)	
> 5	52 (72)	9 (35)	17 (61)	10 (29)	
Grade					0.0014
I	4 (6)	6 (23)	8 (29)	13 (38)	
II	36 (50)	12 (46)	10 (36)	16 (47)	
III-IV	32 (44)	8 (31)	10 (36)	5 (15)	
Stage					< 0.0001
I-II	13 (18)	8 (31)	16 (57)	29 (85)	
IIIA-IV	59 (82)	18 (69)	12 (43)	5 (15)	
*p53 *mutation					0.0099
No	22 (36)	12 (52)	15 (65)	19 (70)	
Yes	39 (64)	11 (48)	8 (35)	8 (30)	
*β-catenin *mutation					0.0024
No	61 (92)	24 (92)	23 (85)	20 (65)	
Yes	5 (8)	2 (8)	4 (15)	11 (35)	
ETR					< 0.0001
No	17 (26)	11 (46)	19 (70)	29 (88)	
Yes	48 (74)	13 (54)	8 (30)	4 (12)	
5-year survival					0.0003
Yes	14 (19)	8 (31)	15 (54)	19 (56)	
No	58 (81)	18 (69)	13 (46)	15 (44)	

Abbreviation: +, present; -, absent

**Figure 3:**
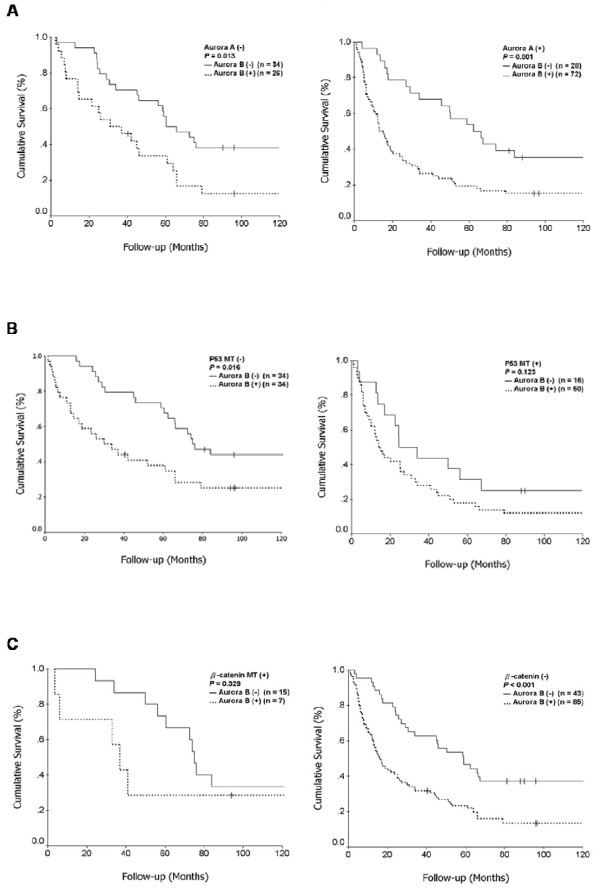
**Cumulative survival curve for HCC in relation to increased or normal expression of *Aurora B *and the presence or absence of *Aurora A *overexpression (A), *p53 *mutation (B), and *β-catenin *mutation (C)**.

Because *Aurora B *overexpression was correlated with *Aurora A *overexpression (*P *= 0.0003), *p53 *mutation (*P *= 0.002), and infrequent *β-catenin *mutation (*P *= 0.002) in this study (Table 1), we then analyzed the prognostic value of *Aurora B *overexpression for patient survival in relation to *Aurora A *overexpression and *p53/β-catenin *mutations. We showed that HCC with *Aurora B *overexpression was associated with worse 5-year survival regardless of *Aurora A *expression status (*P *= 0.013 for HCC without *Aurora A *overexpression and *P *= 0.001 for HCC with *Aurora A *overexpression; Fig. 3A), *p53 *mutation (*P *= 0.016 in wild-type *p53 *HCC and *P *= 0.123 in *p53*-mutated HCC; Fig. 3B), and *β-catenin *mutation (*P *= 0.329 in *β-catenin*-mutated HCC and *P *< 0.001 in wild-type *β-catenin *HCC; Fig. 3C).

### Anticancer effects of *Aurora B *kinase selective inhibitor, AZD1152-HQPA, in HCC cells

**Figure 4:**
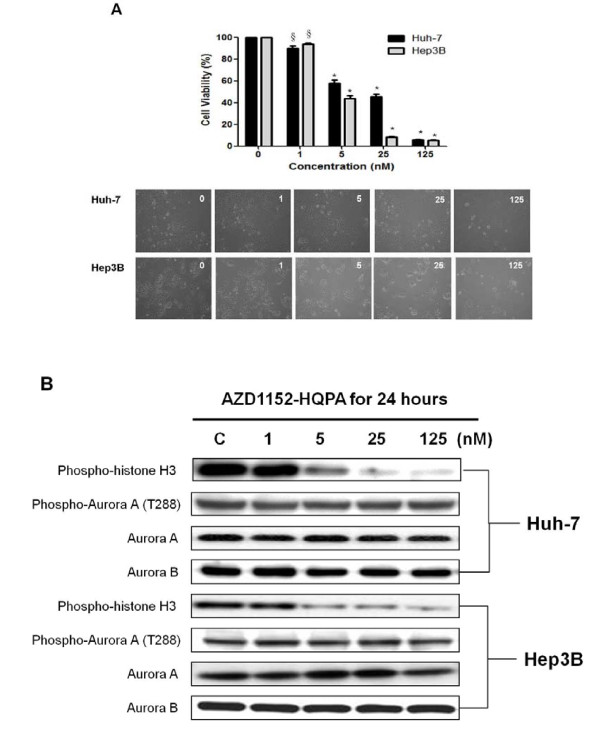
**Aurora B kinase selective inhibitor, AZD1152-HQPA, shows antiproliferative effects in HCC cells**. (A) Concentration-dependent inhibitory effects of AZD1152-HQPA on cell viability in Huh-7 and Hep3B cell lines. Cells were treated with AZD1152-HQPA at 1, 5, 25, and 125 nM in 10% FBS-supplemented medium for 72 hours; cell viability was determined by trypan blue assay. *Columns*: means; *bars*: SD (*n *= 3). §, *p *< 0.05; *, *p *< 0.01 (compared with untreated controls). The changes in cell counts and morphologic features are shown in the lower panels (magnification, × 100). (B) Huh-7 and Hep3B cells were treated with AZD1152-HQPA for 24 hours. The cell lysates were then immunoblotted. Histone H3 (Ser10) phosphorylation, the key substrate of Aurora B signaling, was downregulated in a concentration-dependent manner. Aurora A (T288) phosphorylation, the key substrate of Aurora A signaling, was not repressed by AZD1152-HQPA treatments.

The association of *Aurora B *overexpression and tumor invasiveness of HCC prompted us to explore the effects of Aurora B kinase inhibition on HCC cell viability. Huh-7 and Hep3B cells were treated with increasing concentrations of an Aurora B selective small-molecule inhibitor, AZD1152-HQPA, for 72 hours. Concentration-dependent inhibitory effects of cell viability were observed in both cell lines (Fig. 4A). The ratios of viable Huh-7 and Hep3B cells consistently decreased with higher concentrations of AZD1152-HQPA. The 50% inhibitory concentrations of cell viability (IC_50_) at 72-hr were 16.72 ± 2.44 nM and 4.79 ± 1.03 nM for Huh-7 and Hep3B, respectively (Fig. 4A). Aurora A autophosphorylation at T288 [29] and histone H3 phosphorylation at Ser10 [12] represent the activity of Aurora A and Aurora B, respectively. As shown in Fig. 4B, AZD1152-HQPA induced dephosphorylation of histone H3 (Ser10) in a concentration-dependent manner, while the phosphorylation level of Aurora A (T288) did not change. The data suggest that AZD1152-HQPA exerts its anticancer effects in HCC cells through the inhibition of Aurora B.

**Figure 5:**
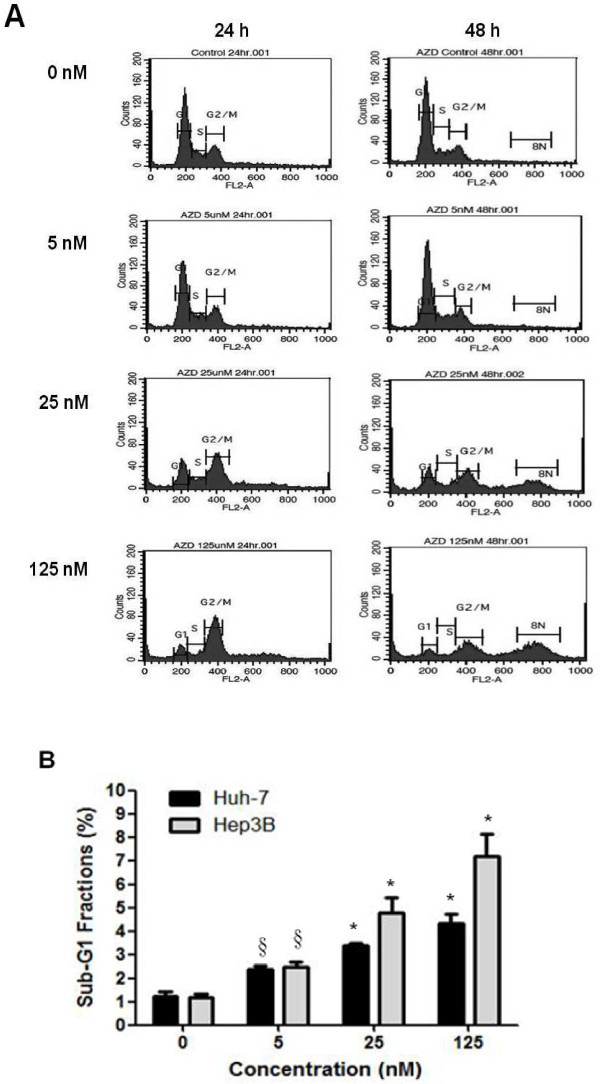
**AZD1152-HQPA treatment leads to cell cycle arrest, accumulation of cells with ≥4*N *DNA content, and apoptosis in HCC cells**. (A) Hep3B cells were treated with vehicle or AZD1152-HQPA at 5, 25, and 125 nM for 24 to 48 hours, and then stained with propidium iodide. DNA contents were analyzed by flow cytometry. Data shown are representative of three independent experiments. (B) Percentage of sub-G1 DNA contents in Huh-7 and Hep3B cells treated with vehicle or AZD1152-HQPA at 5, 25, and 125 nM for 48 hours. *Columns*: means; *bars*: SD (*n *= 3). §, *p *< 0.05; *, *p *< 0.01 (compared to untreated controls).

Because Aurora kinase inhibitors have been shown to induce cell death after the cell cycle has been disturbed [10]. We therefore investigated the effects of AZD1152-HQPA on HCC cell cycle progression and apoptosis. As shown in Fig. 5A, AZD1152-HQPA treatment resulted in accumulation of Hep3B cells with 4N DNA contents at 24-h, followed by the appearance of cells with 8N DNA contents at 48-h. Our data demonstrated that AZD1152-HQPA induced cell cycle disturbance in a concentration-dependent manner (Fig. 5A). We also examined the ability of AZD1152-HQPA to induce apoptosis in HCC cells. As shown in Fig. 5B, AZD 1152-HQPA induced concentration-dependent apoptosis in both Huh-7 and Hep3B cells. After 48 hours of treatment with AZD1152-HQPA above 25 nM, the sub-G1 fractions of Huh-7 cells and Hep3B cells significantly increased (*P *< 0.01). In Fig. 5B, AZD1152-HQPA induced apoptosis more efficiently in Hep3B cells, which is in accordance with the antiproliferative effects.

## Discussion

In mammals, there are three highly related Aurora kinases: Aurora A, B, and C. These 3 closely related kinases share a high degree of sequence homology in their catalytic domains [30]. Despite the sequence homology and common association with mitotic regulatory events, the subcellular localization and signaling substrates differ, and hence the functions of Aurora A and Aurora B are essentially distinct [13]. We have reported that Aurora A is highly expressed in HCC and that overexpression is closely associated with aggressive tumor phenotypes and worse patient prognosis [11], but the clinicopathologic significance of Aurora B in HCC progression remains to be clarified. In this study, we demonstrated that overexpression of *Aurora A *and *Aurora B *was detected in 63% and 61% of 160 surgically resected, primary unifocal HCCs, respectively. Importantly, *Aurora B *mRNA expression correlated with major clinicopathologic parameters related to tumor progression by univariate analyses, including high AFP level (*P *< 0.0001), large tumor size (*P *= 0.021), higher tumor grade (*P *= 0.0007), and higher tumor stage (*P *< 0.0001). By multivariate analyses, we showed that *Aurora B *overexpression was associated with high-stage (stages IIIA, IIIB, and IV) HCC, which exhibits vascular invasion and various extent of microscopic intrahepatic spread (OR, 7.439; *P *= 0.0003). These findings suggest that overexpression of *Aurora B *is associated with tumor invasion and intrahepatic metastasis of HCC, as having been shown in *Aurora A *[11].

Although the diagnosis and management of HCC have progressed significantly, the prognosis for patients receiving surgical treatment remains poor because of the high ETR [6,31]. Hence, the identification of molecular factors to predict ETR will help develop better strategies for patient management. Here, we showed that HCC with *Aurora B *overexpression had a greater than 2-fold higher chance of ETR than HCC without the overexpression (OR, 8.71; 95% confidence interval [CI], 4.02-18.91; *P *< 0.0001). Consistent with its association with high-stage HCC and frequent ETR, HCC with *Aurora B *overexpression showed worse 5-year survival than those without the overexpression (OR, 4.19; 95% CI, 2.11-8.36; *P *< 0.0001). The multivariate analysis confirmed that *Aurora B *overexpression was an independent risk factor associated with ETR (OR, 4.679; *P *= 0.0011; Table 2). These findings are consistent with the correlation of Aurora B overexpression with poor tumor differentiation and worse patient survival in thyroid [32], prostate [33], and hepatobiliary cancers [34,35]. Taken together, our findings suggest that *Aurora B *overexpression serves as a useful marker predicting ETR and hence poor prognosis.

In the present study, we showed that the expression of *Aurora B *and *Aurora A *were closely correlated (*P *= 0.0003; Table 1) and exhibited an interaction contributing to HCC progression. HCC with overexpression of both kinases exhibited the highest rates of high AFP level (71%), vascular invasion (stage IIIA-IV; 82%), and ETR (74%), 4-fold higher than those without overexpression of either kinase (15%, 15%, and 12%, respectively). Consistently, HCC with overexpression of both kinases showed the lowest 5-year survival (19%), approximately one-third of those without any overexpression (56%). Our findings suggest that Aurora A and Aurora B contribute cooperatively to a more malignant HCC phenotype, ETR, and poor prognosis.

HCC has been classified into two major groups according to chromosomal stability status [36]; tumors characterized by chromosomal instability were associated with more *p53 *mutation and less *β-catenin *mutation, the two major genetic mutations in human HCC [17,24,25]. Mutation of *p53 *correlated with aggressive HCC and poor prognosis [24,25], whereas *β-catenin *mutation was associated with less tumor aggression and more favorable prognosis [17]. We also showed that *Aurora A *overexpression correlated positively with *p53 *mutation and inversely with *β-catenin *mutation [11]. In the present study, we showed that *Aurora B *overexpression positively correlated with *p53 *mutation (*P *= 0.002) and inversely with *β-catenin *mutation (*P *= 0.002). Despite the association with these important molecular factors, *Aurora B *overexpression predicted worse 5-year survival regardless of *Aurora A *expression status, *p53 *mutation, or *β-catenin *mutation (Fig. 3). Hence, it is suggested that *Aurora B *overexpression, independent of *Aurora A *overexpression and *p53*/*β-catenin *mutations, is an important molecular factor associated with vascular invasion, leading to high-stage tumor, ETR, and poor prognosis for patients with surgically resected HCC.

Since the discovery of Aurora kinases, Aurora A has attracted much attention as an appealing therapeutic target because of its oncogenic potential [37], and the frequent overexpression of Aurora A in a variety of human cancers [38]. However, subsequent pharmacologic studies demonstrated that dual Aurora A and Aurora B kinase inhibitors produced biologic responses equivalent to Aurora B disruption alone [10], suggesting that Aurora B is a critical therapeutic target for cancer. We previously reported that a novel dual Aurora A and Aurora B kinase inhibitor, VE-465, had anticancer effects in human HCC [12]. Hence, determining whether Aurora A or Aurora B is the pertinent therapeutic target for HCC is imperative. In the present study, we first showed that *Aurora B *overexpression was associated with major clinical (high AFP, ETR) and histopathologic (large tumor, higher tumor grade, and higher tumor stage) features, which are critical for tumor progression of HCC, and hence is an independent risk factor for poor prognosis of patients with surgically resected HCC. In addition, we showed AZD1152-HQPA, an Aurora B selective inhibitor, has anticancer effects in HCC cells. AZD1152-HQPA treatment resulted in profound inhibition of Aurora B signaling, which in turn led to cell cycle disturbance, apoptosis, and growth suppression in HCC cells. Our results suggest that Aurora B selective inhibitors are potential drugs for HCC treatment, confirming the observation that AZD1152 is a novel promising therapeutic approach for HCC [39]. Nevertheless, whether targeting Aurora B kinase alone is a better therapeutic strategy, compared with the targeting of both Aurora A and Aurora B kinases, will require further exploration.

## Conclusion

In this study, we showed frequent overexpression of *Aurora B *in HCC, which was closely associated with aggressive tumor phenotypes. *Aurora B *overexpression, independent of *Aurora A *overexpression and *p53*/*β-catenin *mutations, is an important molecular marker associated with early recurrence and poor prognosis. Besides, an Aurora B kinase selective inhibitor, AZD1152-HQPA, had anticancer effects in HCC cells. These findings indicate the importance of Aurora B kinase in HCC progression and as a potential therapeutic target for HCC.

## Abbreviations

HCC: hepatocellular carcinoma; AFP: α-fetoprotein; ETR: early tumor recurrence; RT-PCR: reverse transcription-polymerase chain reaction; OR: odds ratio.

## Competing interests

The authors declare that they have no competing interests.

## Authors' contributions

ZZL has made substantial contributions to conception, experimental design, data analysis, and manuscript writing of this study. YMJ conceived and designed the study. FCH was responsible for the statistical analysis. HWP participated in the experimental design. HWT performed the RT-PCR. PLL performed analysis of *p53 *and *β-catenin *mutations. PHL supplied tissue samples and collected clinical data. ALC and HCH participated the conception and design of the study, guided the data analysis, manuscript preparation, and reviewed the manuscript. All authors read and approved the final manuscript.

## Pre-publication history

The pre-publication history for this paper can be accessed here:

http://www.biomedcentral.com/1471-2407/10/461/prepub
